# The Association of Pension Income with the Incidence of Type I Obesity among Retired Israelis

**DOI:** 10.1155/2019/5101867

**Published:** 2019-07-25

**Authors:** Yuval Arbel, Chaim Fialkoff, Amichai Kerner

**Affiliations:** ^1^Sir Harry Solomon School of Economics and Management, Western Galilee College, Acre 2412101, Israel; ^2^Institute of Urban and Regional Studies, Hebrew University of Jerusalem, Mt. Scopus, Jerusalem 9190501, Israel; ^3^School of Real Estate, Netanya Academic College, 1 University Street, Netanya 4223587, Israel

## Abstract

Previous studies have identified obesity and overweight as the fourth leading risk factor for global mortality. The objective of the current study is to investigate gender differences and the impact of wealth and income from pensions, sociodemographic variables, and self-assessment of health conditions on the projected probability to become obese in the postretirement age (67 years and older). We are unaware of previous studies, which explored the direct relationship between obesity, monetary income from pensions, wealth, and self-assessment of health conditions. To conduct this research, we make use of an extensive questionnaire concerning the economic and sociodemographic features and health and housing conditions of individuals administered within the framework of the 2015-2016 longitudinal survey conducted by the Israeli Central Bureau of Statistics (CBS). The survey is representative of the Israeli population and also includes information regarding the weight, height, gender, and age of each household member. Results of our study demonstrate that while for the female respondents older than 67, the projected probability of type I obesity (BMI ≥ 30) *drops* by 0.41% (*p*=0.0021) to 0.52% (*p*=0.0001) with an incremental 10,000 NIS (about $2,500) rise of gross annual income from a pension, for the male respondents above 67 years, the projected probability remains unchanged (*p*=0.4225). This outcome remains robust even when the 2015 BMI measurement of type I obesity (BMI ≥ 30) is controlled. This drop among women attenuates with a cutoff point increase from BMI ≥ 25 (overweight) to BMI ≥ 30 (type I obesity) to BMI ≥ 35 (type II obesity). Further results indicate that for both genders above 67 years and for men above 62 years, the projected BMI drop of one year *decreases* with income from a pension (*p*=0.013, *p*=0.039, and *p*=0.007, respectively), although the spread around the projection becomes wider. Compared with other martial status categories, for widowed females, the projected probability of obesity and self-reporting on improved health conditions *drops* by 6.58% (*p*=0.0419) to 11.28% (*p*=0.0048) and 6.55% (*p*=0.0190) to 7.47% (*p*=0.0036), respectively. For females older than 67, family status divorced *drops* the projected probability of obesity by 9.25% (*p*=0.0319). For males older than 67, results show a *rise* in projected obesity with car ownership by 6.10% (*p*=0.0897) to 6.41% (*p*=0.0469) and a *drop* in projected obesity with academic degree status by 9.93% (*p*=0.0106) to 10.14% (*p*=0.0118) and immigration status from American-European countries by 7.67% (*p*=0.0821) to 8.99% (*p*=0.0398) and Asian-African countries by 11.63% (*p*=0.0245) to 11.99% (*p*=0.02). Research findings stress the differences and similarities in male-female patterns of obesity after the retirement age of 67 years and may be of assistance to welfare and public health experts.

## 1. Introduction

Previous studies have identified obesity and overweight as the fourth leading risk factor for global mortality, responsible for an estimated number of 3.2–5.0 million deaths annually [1, 2]. Recently, Nyberg et al. [3] estimated the loss of disease-free years associated with class II-III obesity (BMI ≥ 35, BMI = Weight ÷ (Height^2^), where Weight is measured in kilograms and Height is measured in meters) to range between 7.1 and 10.0 years in subgroups of participants of different socioeconomic status categories, levels of physical activity, and smoking habits.

Following Arbel et al. [4], the objective of the current study is to investigate gender differences and the impact of wealth and monetary income from pensions, sociodemographic variables, and self-assessment of health conditions on the projected probability to become obese in the postretirement age (above 67 years). Given the public expenses associated with increasing life expectancy in Western countries, the study of this cohort might prove to be important in formulating more focused and effective public policies and programs. To the best of our knowledge, these relationships have not been previously explored in the context of gender differences in the postretirement age. The current literature addresses the impact of retirement and occupation on body weight, while a few studies made use of income categories rather than precise monetary income (e.g., [5–9]).

To conduct this research, we make use of an extensive set of questions concerning the economic and sociodemographic features and health and housing conditions of each respondent asked within the framework of the 2015-2016 longitudinal survey conducted by the Israeli Central Bureau of Statistics (CBS). The survey is representative of the Israeli population and includes information regarding the weight, height, gender, and age of each household member.

A common and accepted measure of classifying bodyweight uses BMI. This investigation uses definitions proposed by the World Health Organization, obesity and overweight. Key facts: overweight (BMI ≥ 25), type I obesity (BMI ≥ 30), and type II obesity (BMI ≥ 35) (available at https://www.who.int/en/news-room/fact-sheets/detail/obesity-and-overweight; accessed at March 28)[10].

Results of our study demonstrate that while for the female respondents above 67 years, the projected probability of type I obesity (BMI ≥ 30) *drops* with an incremental 10,000 NIS (about $2,500) rise of gross annual income from a pension by 0.41% (*p*=0.0021) to 0.52% (*p*=0.0001), for the male respondents above 67 years, the projected probability remains unchanged (*p*=0.4225). This outcome remains robust even when the 2015 BMI measurement of type I obesity (BMI ≥ 30) is controlled. This drop in the female group attenuates with a cutoff point increase from BMI ≥ 25 (overweight) to BMI ≥ 30 (type I obesity) to BMI ≥ 35 (type II obesity). Further results indicate that compared with other martial status categories, for widowed females, the projected probability of obesity and self-reporting on improved health conditions *drops* by 6.58% (*p*=0.0419) to 11.28% (*p*=0.0048) and 6.55% (*p*=0.0190) to 7.47% (*p*=0.0036), respectively. For females older than 67, the family status divorced *drops* the projected probability of obesity by 9.25% (*p*=0.0319).

For males above 67, the outcomes demonstrate a *rise* in projected obesity with car ownership by 6.10% (*p*=0.0897) to 6.41% (*p*=0.0469) and a *drop* in projected obesity with academic degree status by 9.93% (*p*=0.0106) to 10.14% (*p*=0.0118) and immigration status from American-European by 7.67% (*p*=0.0821) to 8.99% (*p*=0.0398) and Asian-African countries by 11.63% (*p*=0.0245) to 11.99% (*p*=0.02).

Finally, for both genders above 67 years and for men above 62 years, a projected BMI drop of one year *decreases* with income from a pension (*p*=0.013, *p*=0.039, and *p*=0.007, respectively), although the spread around the projection becomes wider.

Research findings stress the differences and similarities in male-female patterns of obesity after the retirement age of 67 years and may be of assistance to welfare and public health experts.

The rest of this paper is organized as follows.  Section 2 provides an analytical framework.  Section 3 (Materials and Methods (A): A Comparison between Two Consecutive Years) provides descriptive statistics, presents the empirical model, and reports the results, where the data are arranged to permit testing the BMI change across one year and to control type I obesity (BMI ≥ 30) measured in 2015.  Section 4 (Materials and Methods (B): A Conventional Analysis of Panel Data) applies the analysis to a conventional panel data structure and provides robustness tests where the cutoff point definition of obesity changes from BMI ≥ 25 (overweight) to BMI ≥ 30 (type I obesity) to BMI ≥ 35 (type II obesity). Finally, Section 5 (Discussion) concludes and summarizes.

## 2. Analytical Framework

A long-debated question in the literature is the relationship between mortality rates, health, and wealth, and, in particular, “what do the gaps in health have to do with gaps in income?” ([11], page 113). In an influential study and by logistic curve-fitting based on cross-sectional average data at the country level for the years 1900, 1930, and 1960, Preston [12] demonstrated (1) a positive relationship between life expectancy at birth and GDP per capita at a decreasing pace and (2) an upward curve shift from 1930 to 1960. Following Preston [12], the conventional assumption that income is the most important cause of mortality decline has been an unquestioned starting point. Deaton [13], for example, indicated that “people whose reported family incomes in 1980 were less than $5,000 in 1980 prices are estimated to have a life expectancy around 25 percent lower than those whose family incomes were above $50,000” (page 13). On the contrary, given that many health improvements that have been achieved are relatively inexpensive, Deaton concludes that cross-country mortality differences are not the direct outcomes of income inequality but rather of government institutional efficiency. The same institutional characteristics that make countries good at producing output make them good at providing clean water and access to medical care ([11], page 113). Moreover, in a recent study, Lutz and Kebede [14] have demonstrated that the important explanatory variable is the education level rather than the income level.

Referring specifically to the BMI measure and obesity, Subramanian et al. [15], who explored cross-country differences in height, found a strong positive association between height and household wealth. The authors conclude that “Socioeconomic inequalities in height remain persistent. Height has stagnated or declined over the last decades in low- to middle-income countries, particularly in Africa, suggesting worsening nutritional and environmental circumstances during childhood” (quoted from the abstract of the article). Jolliffe [16] addresses the claim that according to the United States National Health and Nutrition Examination Survey (NHANES) data, the null hypothesis of the same overweight rates across income levels cannot be rejected at any time in the last 35 years. Given the nonsymmetrical BMI distribution, the author uses quantile regression and demonstrates that for those at the tails of the BMI distribution, increases in income are correlated with healthier BMI values. [17] demonstrate that at a macrolevel of OECD countries, excluding USA and Mexico (which lead the obesity chart and exhibit obesity rates of 40%), no association was found between obesity and income inequality in OECD countries.

Finally, Zagorsky [18] found a 12% (7%) *decrease* of projected net worth of white (black) women with 1-unit increase in BMI. Lee [19] demonstrated that for both genders at the 51–64-year-old cohort in the USA, the shift from zero or negative net worth to positive net worth is associated with a *decrease* in the projected probability of type I obesity.

## 3. Materials and Methods (A): A Comparison between Two Consecutive Years

The objective of the current section is to explore gender differences and the relationship between BMI change and income from a pension. The data are arranged in a way that each variable is related to one specific year, (either 2016 or 2015). On the one hand, this data structure enables the following:

1. Testing the relationship between BMI2016 and BMI2015 (the BMI of the same person in two consecutive years).
2. Calculating the percent of change as BMI_PER=100 · [(BMI2016/BMI2015) − 1].
3. Testing the difference between female and male BMI distribution for 2016 and 2015.
4. Analyzing the relationships between BMI_PER, INCPENS2016, and BMI30_2015, a dummy variable that equals one where BMI2015 ≥ 30 (type I obesity according to Nyberg et al. [3]) and zero otherwise.

On the other hand, this data structure accounts only for individuals with BMI information on both 2016 and 2015 waves. Consequently, in subsequent sections, we apply the analysis to a conventional panel data structure.

### 3.1. Sample and Controls

The data for this study are based on the longitudinal survey carried out by the Israeli CBS in 2014-2015 and 2016. As an OECD member since 2010, Israel is required to conduct such a survey.

The sample of this survey is representative of the Israeli population, which includes all the Israeli households and persons living in nontherapeutic institutions (students in dormitories, assisted living for the elderly, and absorption centers for new immigrants). The definition of the Israeli population excludes prisoners, inhabitants in therapeutic institutions, such as chronic care housing for the elderly, Israeli inhabitants who remained outside the country for at least one year, foreign workers, diplomats, and Bedouins living in scattered settlements in the southern part of Israel.

The common method used to collect the data from the sampled households is face-to-face interviews at the respondents' homes via computerized questionnaires. Each adult household member above 17 years old was interviewed in one of three languages (Hebrew, Arabic, and Russian).

Given that several studies in the field use telephone interviews (e.g., [20, 21]), the face-to-face interview technique provides two main advantages: (1) unlike phone interviews, the respondent is more likely to answer the questionnaire in a nonthreatening environment with reduced tendency to avoid answers; (2) the interviewer can assess the reliability of answers, particularly regarding environmental housing conditions and height and weight of each household member; (3) families who are sampled by the Israeli CBS are required to cooperate by law. Consequently, high cooperation rates are achieved.

The short term follow up (one year) makes the data structure somewhat similar to a cross section. Molina-Garc et al. [22] and Sallis et al. [1, 23] compared between countries, cities, and individuals based on cross-sectional studies. Nevertheless, the use of one-year follow-up still permits the exploration of the relationship between the BMI of the same person in two consecutive years. Consequently, even the one-year follow-up is somewhat (slightly) better than a cross-sectional study.

Table 1 displays the frequencies of the raw sample based on availability of data with respect to the height and weight variables. From these variables, we measure the BMI as Weight ÷ (Height^2^). The relevant cohorts of our study are above 67 years old (the retirement age for Israeli males) and above 62 years old (the retirement age for the Israeli females). As can be seen from the table, information regarding these variables is available for more than 90% of the respondents. The apparent reason for these high cooperation rates is the legal requirement to cooperate by law with the interviewers.

Tables 2 and 3 report the descriptive statistics referring separately to 841 (1,305) respondents above 67 (62) years with weight and height measured in both the 2016 and 2014-2015 waves. As previously noted, the former (latter) group consists of 70.91%–74.79% (74.49%–74.06%) of those who had at least one BMI report either in the 2016 or 2014-2015 waves. Consequently, the information loss associated with the current data structure is 25.21%–29.09% (25.51%–25.94%)—at least one-quarter of the original sample.

The average BMI measured in the 2016 wave is 27.12–27.18 (BMI2016) and that in the 2014-2015 wave is 27.14–27.18 (BMI2015). The average BMI increase within one year is 0.18%–0.28% and the standard deviation is 8.57%–8.60% (BMI_PER). Based on the cutoff points defined by Nyberg et al. [3] of BMI ≥ 25 (overweight), BMI ≥ 30 (type I obesity), and BMI ≥ 35 (type II obesity), 65%–66% are overweight and above (BMI25_2015; BMI25_2016), 23% have type I obesity and above (BMI30_2015; BMI30_2016), and 6%-7% suffer from type II obesity (BMI35_2015; BMI35-2016).

**Figure 1:**
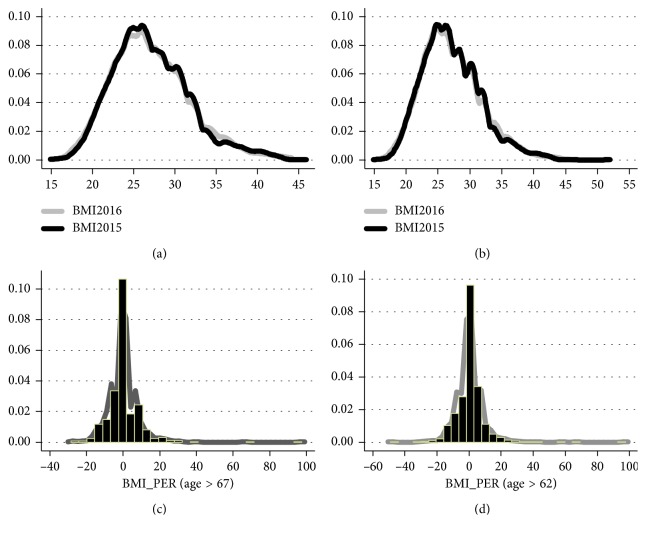
Distribution of BMI2016 vs. BMI2015: pooled sample. Note: the figures refer to the pooled sample of 841 (1,305) women and men above 67 years (above 62 years) with available information on BMI2016 and BMI2015. Given that the retirement age for men (women) in Israel is 67 years (62 years), these ages were chosen as the cutoff points. BMI_PER equals 100 · [(BMI2016/BMI2015) − 1]. For the above 67 years cohort (above 62 years cohort), the null hypothesis, average(BMI2016) = average(BMI2015) cannot be rejected. The *p* value is 0.6025 (0.4998).

Figure 1 displays the distribution of BMI2016 and BMI2015 on the same graph and the distribution of BMI differences in percentage points. The null hypothesis average(BMI2016) = average(BMI2015) cannot be rejected. The respective *p* values for the above 62-year-old and 62-year-old cohorts are 0.6025 and 0.4998.

Returning to Tables 2 and 3, four additional variables are age, gender, percentage of individuals with income from pension, and the amount received only for the latter group of individuals. Depending on the lower bound of the age restriction (Age > 62 or Age > 67), the average age in 2015 is 71.29–74.70 and the maximum age is 80 years (Age2015). 52%–53% of the sample is females (Females2015). Finally, of the pooled sample, 36%–38% receive income from a pension (GET_PENSION2016), and the annual amount received is 84,933–86,023 NIS, the local Israeli currency, where 1 NIS ≈ 0.25 US Dollars (INCPENS2016). Given that the average worker earnings is 142,247 NIS [24], incomes from pensions in the sample consist of 59.71%–60.47% of the average annual salary. In this context, currently, the pension system in Israel is compulsory, and the target is to reach 70% of the last salary before retirement based on 2% annual deposit during 35 years of employment. Consequently, and assuming that prior retirement salary is 142,247 NIS, the 59.71%–60.47% is lower than the desired 70%.

**Figure 2:**
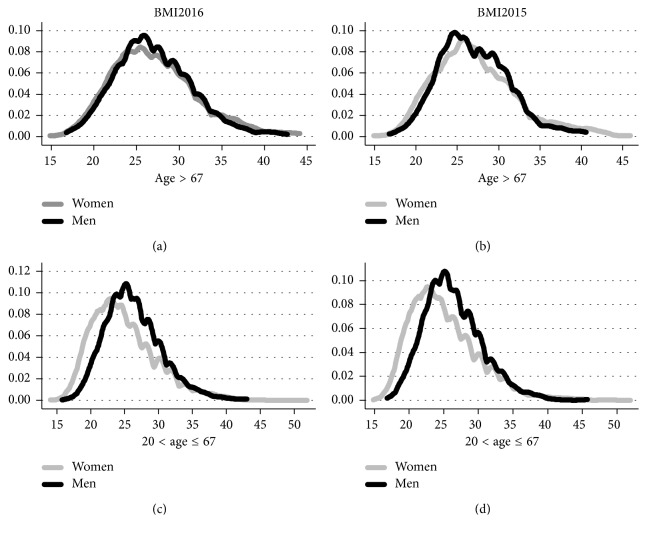
Distribution of BMI2016 and BMI2015 females vs. males. *Note*. The figure compares BMI2016 and BMI2015 of women and men above and below 67 years with available information on BMI2016 and BMI2015. Given that the retirement age for men in Israel is 67 years, this age was chosen as the cutoff point.

Figure 2 displays the distributions of BMI2016 and BMI2015 based on gender differences. We compare the distributions of Age > 67, the postretirement cohorts in 2015, to 20 < Age ≤ 67, the preretirement cohorts for both genders in 2015. To test the male-female BMI differences, we run a Kolmogorov–Smirnoff (K-S) test. The calculated statistics for this test is the maximum BMI difference between the male-female distributions. Results of this test show that with the exception of the Age > 67 group and 2016 measure (*p*=0.165), for all the remaining groups, the maximum BMI difference between the male-female distributions is statistically different from zero (*p*=0.026 and *p* < 0.0001, respectively) Table 4. 

As an interim summary, we have demonstrated that the average BMI measure in 2016 and 2015 is equal, and there are BMI gender differences. The next step is to examine the relationship between the BMI variables and the INCPENS (income from pension) variable.

### 3.2. The Empirical Model

Consider the following independent equations estimated separately for above 67-year-old and above 62-year-old males and females:

(1) $$\begin{array}{c} \text{BMI}2016=\alpha_{0}+\alpha_{1}\;\mathrm{BMI}30\text{\_}2015+\alpha_{2}\left(\text{INCPENS}2016\;÷\;\left(10^{4}\right)\right)+\alpha_{3}\;\mathrm{BMI}30\text{\_}2015\cdot \left(\text{INCPENS}2016\;÷\;\left(10^{4}\right)\right)+\mu_{1}, \end{array}$$

(2) $$\begin{array}{c} \text{BMI\_PER}=\beta_{0}+\beta_{1}\;\mathrm{BMI}30\text{\_}2015+\beta_{2}\left(\text{INCPENS}2016\;÷\;\left(10^{4}\right)\right)+\beta_{3}\;\mathrm{BMI}30\text{\_}2015\cdot \left(\text{INCPENS}2016\;÷\;\left(10^{4}\right)\right)+\mu_{2}, \end{array}$$

where BMI2016 and BMI_PER are the dependent variables; BMI30_2015 and INCPENS2016  ÷  (10^4^) are the independent variables; *α*_0_, *α*_1_, *α*_2_, *α*_3_ and *β*_0_, *β*_1_, *β*_2_, *β*_3_ are parameters; and *μ*_1_, *μ*_2_ are the classical random disturbance terms.

### 3.3. Results

**Figure 3:**
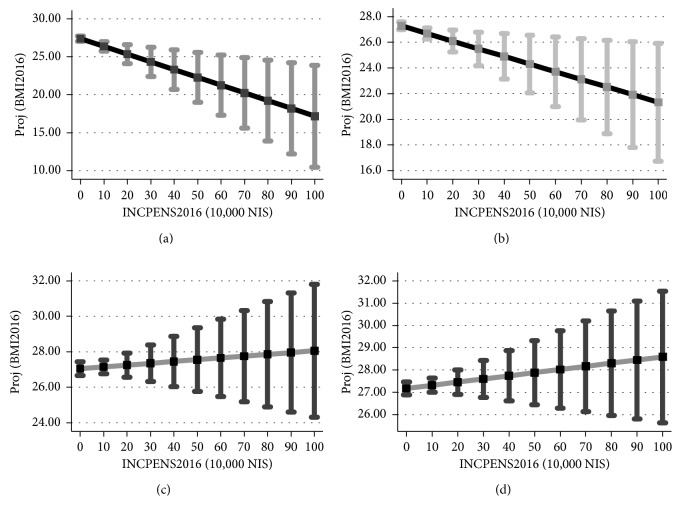
Projected BMI vs. income from a pension in 2016. *Note*. The figure is based on the regression outcomes available upon request based on the model given by equation (1). The vertical axis in each graph is the projected BMI measured in 2016 and the 95% confidence interval for each level of income from a pension. The horizontal axis is income from a pension, measured in 10,000 NIS (10 = 100,000 NIS; 100 = 1,000,000 NIS). NIS is the local Israeli currency (1 NIS ≈ 0.25 US dollars). (a) Women above 67 years. (b) Women above 62 years. (c) Men above 67 years. (d) Men above 62 years.

Figure 3 shows the projected BMI in 2016 as a function of income from a pension based on the estimation of equation (1). Only for women, the projected BMI *drops* with income from a pension (*p*=0.022 for females above 67 years and *p*=0.049 for females above 62 years). (Based on the recommendations of the American Statistical Association (ASA), throughout the article we refrain from using the terminology “statistically [in]significant” and report instead calculated *p* values (see [25, 26] and Wasserstein et al. [27] and, in particular, the recommendation given on page 12. We thank an anonymous reviewer for directing our attention to this issue.) As the figure demonstrates, the spread around the projection becomes wider with higher income from a pension, indicating more heterogeneity in the BMI measure (BMI between 16.75 and 25.90 for above 62-year-old women with annual income from a pension of 1 million NIS). As for men, the projected BMI remains unchanged with income from a pension (*p*=0.908 for males above 67 years and *p*=0.566 for males above 62 years).

**Figure 4:**
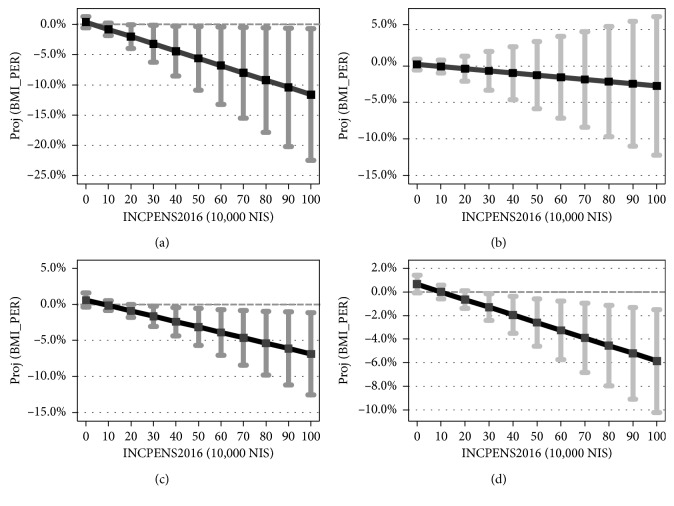
Percentage of BMI change across one year (2015-2016). *Note*. The figure is based on the regression outcomes available upon request based on the model given by equation (2). BMI_PER equals 100 × [[BMI2016/BMI2015] − 1]. (a) Women above 67 years. (b) Women above 62 years. (c) Men above 67 years. (d) Men above 62 years.

Figure 4 shows the projected BMI change during one year as a function of income from a pension based on the estimation of equation (1). The graph also presents the 95% confidence interval around each projection. Based on the graph data, Tables 5 and 6 provide numerical examples for 62-year-old women and men. Results demonstrate that, on the one hand, for both genders above 67 years and for men above 62 years, the projected BMI change *drops* with higher income from a pension (*p*=0.013, *p*=0.039, and *p*=0.007, respectively). On the other hand, the spread around the projection becomes wider with higher income from a pension, indicating more heterogeneity in BMI change (between 0.70% and 22.53% drop for above 67-year-old women with annual income from a pension of 1 million NIS).

Tables 5 and 6 demonstrate that there are no contradictions between Figures 3 and 4:

As can be seen from this example, both BMI2016 and percent of BMI change *drop* with the shift from no annual income to 1 million NIS annual income from a pension.

As can be seen from this example, while BMI2016 *rises* with the shift from no annual income to 1 million NIS annual income from a pension, the percent of BMI change *drops* with the shift from no annual income to 1 million NIS annual income from a pension.

## 4. Materials and Methods (B): A Conventional Analysis of Panel Data

### 4.1. Descriptive Statistics

Tables 7 and 8 present the descriptive statistics of the pooled sample after arrangement based on the conventional panel data structure. The table refers to 1,771 respondents × years. The respondents × years are distributed to 921 (850) females × years (males × years) belonging to 507 (466) households, where the age of both genders was restricted to those older than 67 years, participating in the 2015-2016 longitudinal survey carried out by the Israeli CBS. The lower age bound is based on the retirement age from the workforce, which is 67 years old for males.

According to the OECD report referring to Israel, life expectancy at birth is 82.7 years, compared to 80.9 years in the OECD countries, and life expectancy at age 65 years is 20.6 additional years, compared with 19.7 additional years in the OECD countries. The direct implication from these figures is the required public finance of 20 years after retirement. The age for eligibility for a pension has been increasing gradually since 2004 with increases from 65 to 67 years for men and from 60 years to 62 years for women. Men's retirement age reached 67 years in 2009 while women's is currently 62 and projected to increase to 64 by 2022. Moreover, the percent of working-age population above 65 years is 21.1%, compared with 27.9% in OECD countries [24].

Referring to the frequency of females × years vs. males × years in the sample for different cohorts, at the older cohort of Age > 67, of the 1,771 respondents × years, females × years (males × years) consists of 52% (48%). The proportion of females × years is not different from 50% (*p*=0.0916 and 99% confidence interval of [48.95%, 55.06%]). By comparison, at the younger cohorts of 20 < Age ≤ 67, of the 9,329 respondents × year in the sample, the respective proportions drop (rise) to 50.49% (49.51%) for females × years (males × years). Once again, the proportion of females × years is not different from 50% (*p*=0.3461 and 99% confidence interval of [48.41%, 50.57%]). If the sample is representative, a possible interpretation is slightly lower (higher) mortality rates among females (males). It is also noteworthy that of the total population of adult respondents × year above 20 years, the respondents × year whose age is above 67 consists of 1,771/(1,771+9,329) ≈ 15.95%. The equivalent national ratio for individuals whose age is above 65 years is 10.4% [28].

An acceptable measure of overweight is BMI ≥ 25 and obesity is BMI ≥ 30. As previously noted, BMI is Weight ÷ (Height^2^) where Weight is measured in kilograms and Height is measured in meters. The sample mean BMI reported in Tables 7 and 8 is 26.992 (27.138) for females × years (males × years). Of the 921 (850) females × years (males × years), the frequency of BMI ≥ 30 is 23.0% (21.5%). The 1.5% female-male difference has *p*=0.4519 (BMI30).

**Figure 5:**
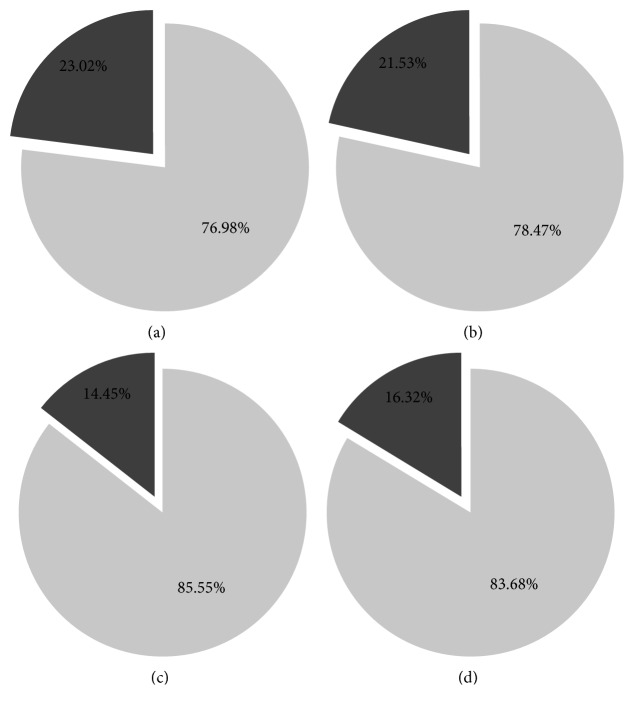
Proportion of obese females and males with BMI ≥ 30 for different cohorts. *Note*. The dark (bright) segment is the proportion of the sample with BMI ≥ 30 (BMI < 30). (a) Females above 67 years. (b) Males above 67 years. (c) Females for whom 20 < Age ≤ 67 years. (d) Males for whom 20 < Age ≤ 67 years.

Figure 5 displays the proportions of obese females and males with BMI ≥ 30 for different cohorts. The figure demonstrates that obesity *rises* with age. As previously noted, among women (men) above 67 years, the frequency of obesity is 23.02% (21.53%). The 1.49% difference across gender has *p*=0.4519, where the 95% confidence interval is [−5.37%, 2.39%]. By comparison, among the women (men) for whom 20 < Age ≤ 67, the frequencies of obesity *drop* to 14.45% (16.32%). The −1.87% difference across gender has *p*=0.0145, where the 95% confidence interval is [−3.38%, −0.37%]. Finally, the *rise* of obesity by 8.57% (5.21%) with age for the group of females (males) has *p* < 0.001.

Referring only to income from pensions, of the 921 (850) female (male) respondents above 67 year, 40.1% (38.0%) receive pensions (GET_PENSION). By comparison to the national level, 46% of the elderly have pensions, and the income replacement ratio in Israel—the after-post pension income ratio—is only 55%–60% as compared to the average of 70%–80% in European countries [28] et al. The average annual pension of the 369 (323) female (male) respondents who receive pension is 74,476 NIS (97,218 NIS). The 22,742 NIS difference has *p*=0.0011, where the 99% confidence interval is [4,757; 40,729] (INCPENS). Given that the average worker earning is 142,247 NIS ([24]), for the females (males) respondents, incomes from pensions consist of 52.36% (68.34%) of the average annual salary.

**Figure 6:**
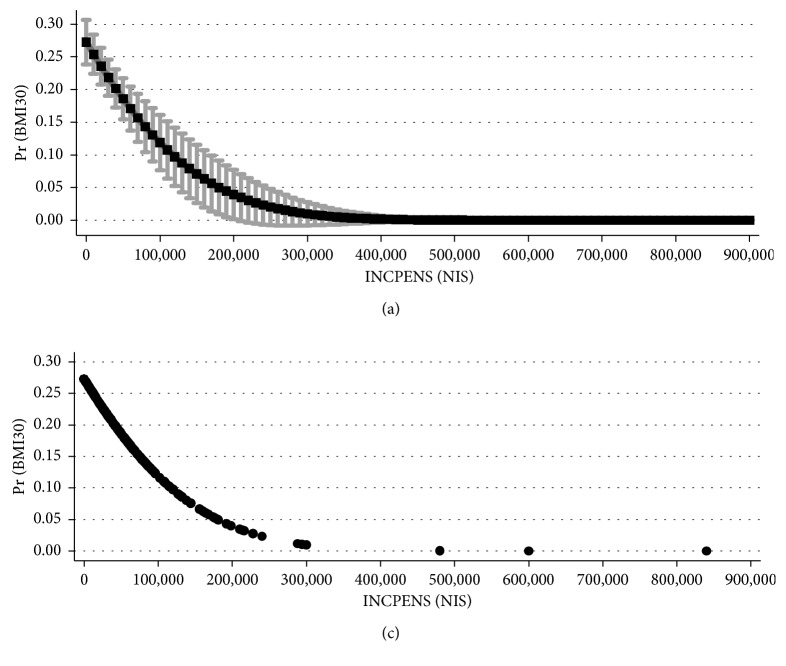
Relationship between projected probability of obesity (BMI ≥ 30) and annual gross income from a pension among females above 67 years. *Note*. The figure describes the projected probability obtained from the probit model, where the dependent variable is BMI30 and the independent variable is INCPENS, the annual gross monetary income from pensions measured in NIS, which refers to the 40.1% (59.9%) of the 921 female respondents above 67 years who got pension (without pension). For the female group, the Pearson correlation between BMI30 and INCPENS (−13.55%) is negative and different from zero correlation (*p* < 0.0001). In contrast, the null hypothesis of zero correlation between BMI30 and INCPENS cannot be rejected for the 850 male respondents (*p*=0.5680).

Figure 6 exhibits the relationship between the projected probability of obesity (BMI ≥ 30) and annual gross income from a pension among females above 67 years. The projected probabilities were obtained from the probit model, where the dependent variable is BMI30 and the independent variable is INCPENS and refers to the 40.1% (59.9%) of the 921 female respondents above 67 years who received a pension (without pension). The graph clearly indicates the drop in the projected probability of obesity from 27.27% for female respondents without pensions to about 3.93% for female respondents with annual income from a pension of 200,000 NIS (about $50,000).

Tables 9 and 10 report the Pearson correlation matrix between the variables BMI30 INCPEN, Books, and Academic. For the female group, the Pearson correlation between BMI30 and INCPENS (-13.55%) is negative and different from zero correlation (*p* < 0.0001). In contrast, the null hypothesis of zero correlation between BMI30 and INCPENS cannot be rejected for the 850 male respondents (*p*=0.5680). The latter outcome implies that this drop with income is unique to females. No such drop is supported empirically among groups of males.

Referring to other wealth, income, and education variables in Tables 7 and 8, of the 921 (850) females × years (males × years), 75.4% (73.2%) live in a housing unit owned by the household (OWNER). By comparison, the national Israeli 2016 average shows that 67.6% are homeowners [29]. Of the 921 (850) females × years (males × years), 97.2% (98.0%) have at least one book in the home library (Books). 46.4% (65.2%) own a car (Car). By comparison, the national 2015 average shows that 69.7% of the 2.4139 million households own at least one car and 24.4% own two cars or more (Israeli CBS, [30]). Finally, 30% (34.2%) hold a BA, MA, or Ph.D. degree from academic institutions (Academic).

According to the reports in Tables 9 and 10, for females, BMI30 is negatively correlated with INCPENS, and this correlation is different from zero correlation (*p* < 0.0001). For both genders, BMI30 is negatively correlated with Academic (*p*=0.0130 for females and *p*=0.0002 for males). The implication is that the projected probability to become obese *drops* with academic education. For both genders, the Pearson correlations between INCPEN and Academic are all positive and different from zero correlation (*p* < 0.0001 for females and *p*=0.0001 for males). As expected, income from pensions *rises* with academic education.

Referring to the quantitative sociodemographic variables in Tables 7 and 8, the sample mean of the age variable is 74.64 for females and 74.66 for males. Given the imposed cutoff point, the minimum age for both genders is 68 years and the maximum age is 80 years (Age). By comparison, according to 2018 Statistical Abstract of Israel ([31]), the median age of the overall Israeli population is 31.0 years (29.8 years) for females (males). The number of household members is about two for both genders (HHSIZE).

Referring to the binary sociodemographic variables in Tables 7 and 8, for the cohort of above 67 years, 3.90% (1.90%) of the female (male) participants are single who were never married (Single), 47.7% (75.1%) are married (Married), 10.2% (8.4%) are divorced (Divorced), and 38.2% (14.7%) are widowed (Widow). By comparison, according to the 2018 Statistical Abstract of Israel ([32]), the total Israeli population in 2016 includes 3.1317 (2.9987) million females (males), of whom 0.2451 ÷ 3.1317 = 7.826% (0.0524 ÷ 2.9987 = 1.747%) are a widow (widower). Once again, the implication from these data might be that compared with females, mortality rates among males are higher. Indeed, the national statistics show that life expectancy of women is longer −84.2 years for females and 80.4 years for males (Israeli Central Bureau of Statistics Press Release, at [33]).

Finally, 71.3% (69. 6%) are female (male) immigrants (Immigrants), of whom 47.7% (47.1%) are from European-American countries (IMM_EUROPE_AMERICA) and 23.7% (22.6%) from Asian-African countries (IMM_ASIA_AFRICA). These figures imply that 66.8% (67.6%) of the female (male) immigrants are from European-American countries (IMM_EUROPE_AMERICA_PER). By comparison, according to the 2018 Statistical Abstract of Israel, the proportion for the entire population of immigrants who emigrated to Israel in 1948–2017 in favor of European-American immigrants is 70.1% [34].

An interesting psychological feature in our sample is the self-assessment of overall health. Of the 921 (850) females × years (males × years), 48.4% (54.8%) reported on good overall health. Stratification by cohorts show that compared with the adult group (Age > 67), the report on good health among young respondents (20 < Age ≤ 67) *rises* by 36.99%, *p* < 0.0001 (32.13%, *p* < 0.0001). Indeed, in the national level, compared with the younger cohorts, the self-assessment of health conditions among the older cohorts tends to be lower, and compared with women, the self-assessment of men tend to be higher [28].

### 4.2. The Empirical Model

Consider the following empirical model estimated separately for female and male respondents for the pooled sample:

(3) $$\begin{array}{c} \text{BMI}30=\gamma_{0}+\gamma_{1}\left(\text{INCPENS }÷\;\left(10^{4}\right)\right)+\gamma_{2}\text{OWNER}+\gamma_{3}\text{BOOKS}+\gamma_{4}\text{CAR}+\gamma_{5}\text{AGE}+\gamma_{6}\text{ACADEMIC}+\gamma_{7}\text{HHSIZE}+\gamma_{8}\text{MARRIED}+\gamma_{9}\text{DIVORCED}+\gamma_{10}\text{WIDOW}+\gamma_{11}\text{IMM\_EUROPE\_AMERICA}+\gamma_{12}\text{IMM\_ASIA\_AFRICA}+\gamma_{13}\text{OVERALL\_HEALTH}+D{\vec{\delta }}_{1}+\mu_{3}, \end{array}$$

where BMI30, the dependent variable, is a dummy variable that equals 1 for obesity (BMI ≥ 30) and 0 otherwise. The independent variables include (1) wealth, income, and education proxies: INCPENS, Owner, Books, Car, and Academic; (2) quantitative sociodemographic characteristics: Age and HHSIZE; (3) family-status variables: Married, Divorced, and Widow (base category of Single); (4) immigration variables: IMM_EUROPE_AMERICA and IMM_ASIA_AFRICA (base category of Native Israeli); and (5) self-assessment of overall health OVERALL_HEALTH. *α*_0_, *α*_1_,…, *α*_13_ are parameters, *D* is a matrix of individual effect dummies, *δ*_1_ is a column vector of parameters, and *μ*_1_ is the stochastic random disturbance term.

### 4.3. Results

Table 11 reports the estimation results of the random effect panel regressions given by equation (3) among females and males above 67 years. The dependent variable is BMI30, a dummy variable that equals 1 for obesity, and 0 otherwise (BMI ≥ 30). This Linear Probability Model (LPM) yields projected probabilities of obesity; see the discussions in [35]: 415–418 and [36]: 727–730. Following the F-statistics, which reject the null hypothesis that the coefficients of individual effect dummies are equal, all the regressions include dummies Fixed-Effects, otherwise the coefficients might be inefficient (for a discussion see [35]: 391–395; [36]: 386–387). To correct for the inherent heteroskedasticity associated with the LPM, robust *p* values are given in parentheses (for a discussion concerning robust standard errors see [35]: 162–166; 415–418; [36]: 428–429; 727–730). To provide the full information, the odd columns report the full model (e.g., [25, 26]. The even columns report the stepwise model, which is obtained by gradual omission of variables with coefficients for whom *p* < 0.05.

Results for women above 67 years indicate that the projected probability to become obese *drops* by 0.41% (*p*=0.002) to 0.52% (*p*=0.001) with each additional 10,000 NIS to the annual gross pension. In contrast, for the male group above 67 years, regardless of the income level from a pension, the projected probability to become obese remains unchanged (*p*=0.4225).

Referring to the female group above 67 years, and compared with married, divorced, and single women, the projected probability to become obese among widowed females above 67 years *rises* by 6.58% (*p*=0.0419) to 11.28% (*p*=0.0048). The projected probability to become obese among divorced females above 67 years *rises* by 9.25% (*p*=0.0319). Female respondents with a BA, MA, or Ph.D. degree are less likely to become obese by 6.23% (*p*=0.0966).

Referring to the male group above 67 years, the projected likelihood to become obese *rises* by 6.10% (*p*=0.0897) to 6.41% (*p*=0.0467) with ownership of at least one car. Male respondents with a BA, MA, or Ph.D. degree are less likely to become obese by 9.93% (*p*=0.0106) to 10.14% (*p*=0.0118). Finally, compared with native Israeli males, the projected odds to become obese *drop* by 7.67% (*p*=0.0821) to 8.99% (*p*=0.0398) for male immigrants above 67 years from American-European countries. Compared with native Israeli males, the projected odds to become obese *drop* by 11.63% (*p*=0.0245) to 11.99% (*p*=0.02) for male immigrants above 67 years from Asian-African countries.

An interesting feature of our study is the self-ranking of overall health conditions. Results of our study demonstrate that for the female group above 67 years, compared with the base category—low self-ranking of health conditions—projected likelihood to become obese *drops* by 6.55% (*p*=0.0190) to 7.47% (*p*=0.0036) with self-ranking of good overall health conditions. In contrast, for the male group above 67 years, the coefficient of OVERALL_HEALTH is found to remain unchanged (*p*=0.2439).

### 4.4. Robustness Tests

Recently, Nyberg et al. [3] estimated the loss of disease-free years associated with class II-III obesity to range between 7.1 and 10.0 years in subgroups of participants of different socioeconomic levels, levels of physical activity, and smoking habits. In line with their study, we run a robustness test, which the cutoff point definition of obesity changes from BMI ≥ 25 (overweight) to BMI ≥ 30 (type I obesity) to BMI ≥ 35 (type II obesity).  Table 7 shows that, as expected, as the benchmark changes from BMI25 to BMI30 and to BMI35, the relative frequency of females × years above this benchmark *drops* from 65.4% to 23.0% to 7.9%. The equivalent frequencies of males × years are 65.4% to 21.5% to 4.5% (Table 8).

**Figure 7:**
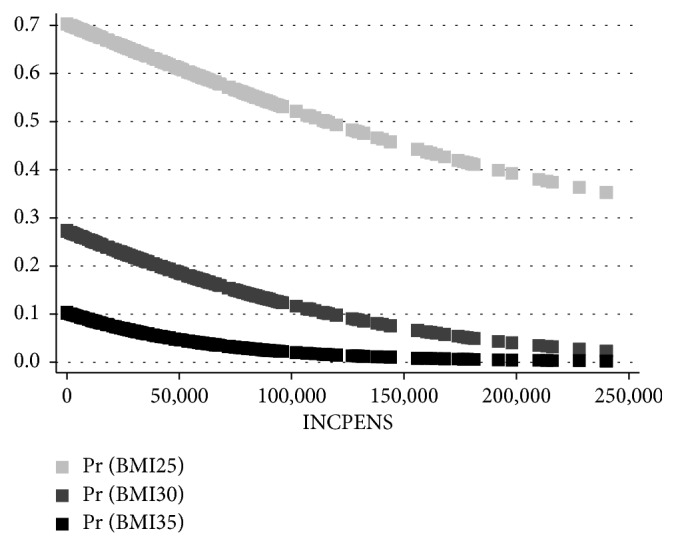
Relationship between the projected probability of Overweight (BMI ≥ 25), Type I (BMI ≥ 30), and Type II obesity (BMI ≥ 35) and annual gross income from pension among females above 67 years. *Note*. The figure describes the projected probabilities obtained from the probit model, where the dependent variables are BMI25, BMI30, BMI35, dummy variables, which equal one for Overweight (BMI ≥ 25), Type I (BMI ≥ 30), and Type II obesity (BMI ≥ 35) and zero otherwise. The independent variable is INCPENS, the annual gross monetary income from pensions measured in NIS (the local Israeli currency, 1 NIS ≈ $0.25), which refers to the 40.1% (59.9%) of the 921 female respondents above 67 years who got pension (without pension). We excluded a few outliers for which INCPES > 250,000. For women, the respective Pearson correlations between BMI25, BMI30, BMI35, and INCPENS (−13.90%, −13.55%, −10.01%) are negative and different from zero correlation (*p* < 0.0001, *p* < 0.0001, and *p*=0.0024, respectively). The Pearson correlations between BMI25, BMI30, BMI35, and INCPENS among the 869 male respondents were found to be equal to zero (*p*=0.5016, *p*=0.5680, and *p*=0.0530, respectively).

Figure 7 describes the projected probabilities obtained from the probit model, where the dependent variables are BMI25, BMI30, BMI35, and dummy variables, which equal one for Overweight (BMI ≥ 25) and Type I (BMI ≥ 30) and Type II Obesity (BMI ≥ 35) and zero otherwise. The independent variable is INCPENS, the annual gross monetary income from a pension measured in NIS (the local Israeli currency, 1 NIS ≈ $0.25), which refers to the 40.1% (59.9%) of the 921 female respondents above 67 years who got a pension (without pension). We excluded a few outliers for which INCPES > 250,000.

As the figure shows, for women, all three benchmarks of obesity exhibit a *drop* with INCPENS. However, an increase in the benchmark from BMI25 to BMI30 to BMI35 attenuates this drop. For women, the respective Pearson correlations between BMI25, BMI30, BMI35, and INCPENS (−13.90%, −13.55%, −10.01%) are negative and different from zero correlation (*p* < 0.0001, *p* < 0.0001, and *p*=0.0024, respectively). The Pearson correlations between BMI25, BMI30, BMI35, and INCPENS among the 869 male respondents were found to be equal to zero (*p*=0.5016, *p*=0.5680 and *p*=0.0530, respectively).

Table 12 presents the Random-Effect Regression 2015-2016: stratification by Overweight (BMI ≥ 25), Type I (BMI ≥ 30), and Type II (BMI ≥ 35) obesity among females above 67 years. The empirical model is based on equation (3), where the dependent variable is replaced from BMI25 to BMI30 and to BMI35. Once again, to provide the full information, the odd columns report the full model (e.g., [25, 26]). The even columns report the stepwise model, which is obtained by gradual omission of variables with coefficients for whom *p* > 0.05.

Results indicate that the projected probability of overweight among retired female respondents *drops* by 0.84% (*p*=0.0056) to 0.95% (*p*=0.0014) with each additional 10,000 NIS annual gross monetary income from a pension. This drop attenuates to 0.41% (*p*=0.0021) to 0.52% (*p*=0.0001) for type I obesity. The projected probability of type II obesity remains unchanged with the annual level of monetary income from a pension (*p*=0.1699).

Interestingly, narrowing the benchmark to type II obesity among retired women modifies the coefficient of Owner to positive. The projected probability of type II obesity *rises* with homeownership by 4.14% (*p*=0.0169) to 4.37% (*p*=0.0089). An additional variable whose coefficient becomes positive is HHSIZE, where the projected probability of type II obesity *rises* by 3.53% (*p*=0.0201) to 3.57% (*p*=0.0211) with each additional person in the household. The projected probability of type II obesity among retired women *rises* by 9.83% (*p*=0.0340) to 12.44% (*p*=0.0572) with family status of divorced and by 5.31% (*p*=0.0377) to 9.06% (*p*=0.0430) with family status of widow. Finally, the sign of the coefficient of OVERALL_HEALTH remains robust where the definition of obesity becomes narrower. The projected probabilities of overweight and type I and type II obesity among retired women *drop* by 2.45% (*p*=0.0721) to 7.47% (*p*=0.0036) with self-report of overall good health conditions.

To simplify the comparison, Table 13 presents the Random-Effect Regression 2015-2016: stratification by Overweight (BMI ≥ 25), Type I (BMI ≥ 30), and Type II (BMI ≥ 35) obesity among males above 67 years. The empirical model is based on equation (3), where the dependent variable is replaced from BMI25 to BMI30 and to BMI35.

Results support the conclusion that regardless of the benchmark, the projected probability remains unchanged with the annual level of monetary income from a pension (*p*=0.4404, *p*=0.4225, and *p*=0.5758, respectively) and with self-reported overall health conditions (*p*=0.9232, *p*=0.2439, and *p*=0.3300, respectively).

Additional outcomes show that for the group of male respondents, the projected probability of type II obesity *rises* with homeownership by 2.55% (*p*=0.0431) to 2.74% (*p*=0.04). Narrowing the benchmark of obesity makes the coefficients of Age closer to zero. The projected probability of overweight decreases with the age variable by 0.99% (*p*=0.045) to 1.03% (*p*=0.0328). However, referring to type I and type II obesity, the coefficient of age remains unchanged (*p*=0.9494 and *p*=0.8297). Also, and with the exception of type I obesity, the coefficients of the variables Car, Academic, and IMM_ASIA_AFRICA remain unchanged with respect to the projected probability of overweight and type II obesity. The coefficient of OVERALL_HEALTH remains consistently unchanged (*p*=0.9232, *p*=0.2439, and *p*=0.33, respectively).

## 5. Discussion

Following Arbel et al. [4], the objective of the current study is to investigate gender differences and the impact of wealth and monetary income from pensions, sociodemographic variables, and self-assessment of health conditions on the projected probability to become obese in the postretirement age—above 67 years. To the best of our knowledge, these relationships have not been previously explored in the context of gender differences in the postretirement age. The current literature addressed the impact of retirement and occupation on body weight, where few of the research studies used income categories rather than precise monetary income (e.g., [5–9]).

To conduct this research, we make use of an extensive questionnaire concerning the economic and sociodemographic features and health and housing conditions of each respondent asked within the framework of the 2015-2016 longitudinal survey conducted by the Israeli CBS. The survey is representative of the Israeli population and includes information regarding the weight, height, gender, and age of each household member.

Methodologically, the article is divided into two sections. In Section 3, the data are arranged to permit testing the BMI change across one year and to observe whether the BMI measured in 2016 is related to income from a pension as well as the frequency of type I obesity (BMI ≥ 30) measured in 2015. The disadvantage of this data structure is information loss associated with individuals without BMI information on both 2016 and 2015 waves. Consequently, in Section 4, we apply the analysis to a conventional panel data structure.

The findings in Section 3 demonstrate that, on the one hand, for women, the projected BMI *drops* with income from a pension (*p*=0.022 for females above 67 years and *p*=0.049 for females above 62 years). On the other hand, the spread around the projection becomes wider with higher income from a pension, indicating more heterogeneity in the BMI measure (BMI between 16.75 and 25.90 for women older than 62 with annual income from a pension of 1 million NIS). As for men, the projected BMI remains unchanged with income from a pension (*p*=0.908 for males above 67 years and *p*=0.566 for males above 62 years).

Further results from Section 3 demonstrate that, on the one hand, for both genders above 67 years and for men above 62 years, the projected BMI change *drops* with higher income from a pension (*p*=0.013, *p*=0.039, and *p*=0.007, respectively). On the other hand, the spread around the projection becomes wider with higher income from a pension, indicating more heterogeneity in BMI change (between 0.70% and 22.53% drop for above 67-year-old women with annual income from a pension of 1 million NIS).

The findings in Section 4 show once again that compared with individual retired women without a pension (a majority of 59.9%), the projected probability of overweight and type I obesity *decreases* by 0.41% (*p*=0.0021) to 0.95% (*p*=0.0001) with an incremental increase of 10,000 NIS. The implication is that wealthier retired female respondents suffer less from obesity. Once again, no such relationship is found among the male participants (*p*=0.4225). Also, widowed and divorced females seem to suffer more from type I and type II obesity problems. The projected probability for type I obesity *rises* by 9.25% (*p*=0.0319) for divorced and by 6.58% (*p*=0.0419) to 11.28% (*p*=0.0048) for widowed females. The projected probability for type II obesity *rises* by 9.83% (*p*=0.0340) to 12.44% (*p*=0.0572) for divorced and by 5.31% (*p*=0.0377) to 9.06% (*p*=0.0430) for widowed females. These findings are supported by Iecovich et al. [28], who discuss the problem of loneliness among the elderly populations. Finally, unlike retired men, retired women exhibit improved awareness to overall health conditions, particularly in the context of obesity. Women who reported on improved overall health conditions are less likely to suffer from overweight and type I and type II obesity by 2.45% (*p*=0.0721) to 7.47% (*p*=0.0036).

An interesting outcome is obtained when we narrow the definition of obesity from Type I (BMI ≥ 30) to Type II obesity (BMI ≥ 35). For both genders, the projected probability of type II obesity increases by 2.55% (*p*=0.0431) to 4.37% (*p*=0.0089) with homeownership. The implication is that unlike previous outcomes, for both genders, richer individuals who own a housing unit suffer more from type II obesity.

Future research should explore the relationship between wealth sources and liabilities, such as saving accounts, mortgage balance, assigned credit line, and overdraft balance and obesity for the elderly population. This type of research may be of assistance to welfare and public health experts.

## Figures and Tables

**Table 1:** Frequencies of the raw sample with respect to weight and height.

	Available	Unknown	Refusal	Total sample
*Weight2016*				
Above 67 (*N*)	1,196	74	14	1,284
Above 67 (%)	93	6	1	100
Above 62 (*N*)	1,745	97	32	1,874
Above 62 (%)	93	5	2	100

*Height2016*				
Above 67 (*N*)	1,186	94	4	1,284
Above 67 (%)	92	8	0	100
Above 62 (*N*)	1,753	112	9	1,874
Above 62 (%)	94	6	0	100

*Weight2015*				
Above 67 (*N*)	1,131	83	17	1,231
Above 67 (%)	92	7	1	100
Above 62 (*N*)	1,762	105	28	1,895
Above 62 (%)	93	6	1	100

*Height2015*				
Above 67 (*N*)	1,129	97	5	1,231
Above 67 (%)	92	8	0	100
Above 62 (*N*)	1,782	105	8	1,895
Above 62 (%)	94	6	0	100

*Note*. (1) The full sample of the above 67 years old cohort in the 2016 wave (2014-2015 wave) includes 1,284 (1,237) persons. (2) The full sample of the above 62 years old cohort in the 2016 wave (2014-2015 wave) includes 1,875 (1,901) persons. (3) Individuals with “unknown” or “refusal” classifications have been deleted from the sample. (4) Of the 1,186 (1,129) persons above 67 years with available information on BMI in 2016 wave (2014-2015 wave), 841 persons, consisting of 70.91% (74.49%), had available BMI information on both 2016 and 2014-2015 waves. (5) Of the 1,745 (1,762) persons above 62 years with available information on BMI in 2016 wave (2015 wave), 1,305 persons, consisting of 74.79% (74.06%), had available BMI information on both 2016 and 2014-2015 waves.

**Table 2:** Descriptive statistics of 2015-2016 panel: pooled sample above 67 years.

Variables	Description	Obs.	Mean	(SD)
Weight2016	Weight in kg with light clothing and no shoes measured in the 2016 wave	841	74	(14.2)
Height2016	Height in centimeters without shoes measured in the 2016 wave	841	165	(9.0)
BMI2016	Body mass=10,000 · (Weight2016/Height2016^2^)	841	27	(4.8)
Weight2015	Weight in kg with light clothing and no shoes measured in the 2015 wave	841	74	(14.1)
Height2015	Height in centimeters without shoes measured in the 2015 wave	841	165	(9.0)
BMI2015	Body mass=10,000 · (Weight2015/Height2015^2^)	841	27	(4.7)
BMI_PER	(BMI2016/BMI2015) − 1	841	0.18%	(8.6%)
BMI25_2015	1 = BMI ≥ 25; 0 = BMI < 25	841	65%	(48.0%)
BMI30_2015	1 = BMI ≥ 30; 0 = BMI < 30	841	23%	(42.0%)
BMI35_2015	1 = BMI ≥ 35; 0 = BMI < 35	841	7%	(25.0%)
Age2015	Age in years	841	75	(4.4)
Females2015	1 = females; 0 = males	841	53%	(50.0%)
GET_PENSION2016	1 = get pension; 0 = otherwise	841	38%	(68.0%)
INCPENS2016 > 0	Household's annual gross monetary income from pensions in NIS for the household	322	86,023	(91,832.0)

**Table 3:** Descriptive statistics of 2015-2016 panel: pooled sample above 62 years.

Variables	Description	Obs.	Mean	(SD)
Weight2016	Weight in kg with light clothing and no shoes measured in the 2016 wave	1,305	75	(14.5)
Height2016	Height in centimeters without shoes measured in the 2016 wave	1,305	166	(9.0)
BMI2016	Body mass=10,000 · (Weight2016/Height2016^2^)	1,305	27	(4.7)
Weight2015	Weight in kg with light clothing and no shoes measured in the 2015 wave	1,305	75	(14.3)
Height2015	Height in meters without shoes measured in the 2015 wave	1,305	166	(9.0)
BMI2015	Body mass=10,000 · (Weight2015/Height2015^2^)	1,305	27	(4.6)
BMI_PER	(BMI2016/BMI2015) − 1	1,305	0.28%	(8.6%)
BMI25_2015	1 = BMI ≥ 25; 0 = BMI < 25	1,305	66%	(48.0%)
BMI30_2015	1 = BMI ≥ 30; 0 = BMI < 30	1,305	23%	(42.0%)
BMI35_2015	1 = BMI ≥ 35; 0 = BMI < 35	1,305	6%	(25.0%)
Age2015	Age in years	1,305	71	(5.9)
Females2015	1 = females; 0 = males	1,305	52%	(50.0%)
GET_PENSION2016	1 = get pension; 0 = otherwise	1,305	36%	(48.0%)
INCPENS2016 > 0	Household's annual gross monetary income from pensions in NIS for the household	464	84,933	(95,758.0)

*Note*. The sample includes 841 (1,305) individuals for whom both BMI2016 and BMI2015 are available, and with age restriction above 67 years old (above 62 years old), participating in the 2015-2016 longitudinal survey carried out by the Israeli CBS. The lower age bound is based on the retirement age from the workforce, which is 67 years old for males and 62 years for females. NIS is the local Israeli currency, where 1 NIS roughly equals $0.25. Standard deviations are given in parentheses.

**Table 4:** The Kolmogorov–Smirnoff (K-S) test measures the maximum difference between the female-male distributions (*D*). Results of the combined K-S test are given in the following table.

Cohort	Observations	BMI2016	BMI2015
Age > 67	*N* = 841; females = 447; males = 394	*D* = 0.0772 (*p* value = 0.165)	*D* = 0.1017^*∗∗*^ (*p* value = 0.026)
20 < Age ≤ 67	*N* = 4,400; females = 2,214; males = 2,186	*D* = 0.2086^*∗∗∗*^ (*p* < 0.0001)	*D* = 0.2127^*∗∗∗*^ (*p* < 0.0001)

^*∗∗*^ *p* < 0.05 for male-female BMI difference. ^*∗∗∗*^ *p* < 0.01 for male-female BMI difference.

**Table 5:** Numerical example: comparison between 2015 and 2016 BMI of women above 62 years. Consider the following example for women above 62 years (the same numbers that are described at the right-upper part of Figures 3 and 4).

BMI2016	27.27488	21.32663
BMI2015	27.2185830969	21.9268070544
(BMI2016/BMI2015) − 1	0.2068326%	−2.737184%
Annual income from a pension	0 NIS	1 million NIS

**Table 6:** Numerical example: comparison between 2015 and 2016 BMI of men above 62 years. Next, consider the following example for men above 62 years (the same numbers that are described at the right-lower parts of Figures 3 and 4).

BMI2016	27.1695	28.5859
BMI2015	26.9931164766	30.3649305728
(BMI2016/BMI2015) − 1	0.6534389%	−5.858833%
Annual income from a pension	0 NIS	1 million NIS

**Table 7:** Descriptive statistics of 2015-2016 panel for women after retirement.

Variables	Description	Obs.	Mean	(SD)
Weight	Weight in kg with light clothing without shoes	921	69	(12.7)
Height	Height in centimeters without shoes	921	160	(6.3)
BMI	Body mass=10,000 · (Weight/Height)	921	27	(4.9)
BMI25	1 = BMI ≥ 25; 0 = BMI < 25	921	65%	(47.6%)
BMI30	1 = BMI ≥ 30; 0 = BMI < 30	921	23%	(42.1%)
BMI35	1 = BMI ≥ 35; 0 = BMI < 35	921	8%	(27.0%)
GET_PENSION	1 = get pension; 0 = otherwise	921	40%	(49.0%)
INCPENS > 0	Household's annual gross monetary income from a pension in NIS for women who are either the respondent or spouse in the household	369	74,476	(76,904.0)
Owner	1 = own an apartment; 0 = otherwise	921	75%	(43.1%)
Books	1 = At least one book at the home library; 0 = no books at the home library	921	97%	(16.6%)
Car	1 = own a car; 0 = otherwise	921	46%	(49.9%)
Age	Age in years	921	75	(4.4)
Academic	1 = Formal academic education with BA, MA or Ph.D. diploma; 0 = otherwise	921	30%	(45.8%)
HHSIZE	Number of persons in household	921	2	(1.2)
Single	1 = single; 0 = otherwise	921	4%	(19.4%)
Married	1 = married; 0 = otherwise	921	48%	(50.0%)
Divorced	1 = divorced; 0 = otherwise	921	10%	(30.3%)
Widow	1 = widow; 0 = otherwise	921	38%	(48.6%)
Immigrant	1 = immigrant; 0 = otherwise	921	71%	(45.2%)
IMM_EUROPE_AMERICA	1 = immigrant from European or American countries; 0 = otherwise	921	48%	(50.0%)
IMM_ASIA_AFRICA	1 = immigrant from Asian or African countries; 0 = otherwise	921	23%	(42.5%)
IMM_EUROPE_AMERICA_PER	1 = immigrant from European or American countries only for the group of immigrants; 0 = otherwise	657	67%	(47.1%)
OVERALL_HEALTH	1 = Self-reporting of good overall health conditions; 0 = otherwise	921	48%	(50.0%)

**Table 8:** Descriptive statistics of 2015-2016 panel for men after retirement.

Variables	Description	Obs.	Mean	(SD)
Weight	Weight in kg with light clothing and without shoes	850	80	(13.5)
Height	Height in meters without shoes	850	171	(6.9)
BMI	Body mass=10,000 · (Weight/Height)	850	27	(4.2)
BMI25	1 = BMI ≥ 25; 0 = BMI < 25	850	65%	(47.6%)
BMI30	1 = BMI ≥ 30; 0 = BMI < 30	850	22%	(41.1%)
BMI35	1 = BMI ≥ 35; 0 = BMI < 35	850	5%	(20.7%)
GET_PENSION	1 = receives pension; 0 = otherwise	850	38%	(48.6%)
INCPENS > 0	Household's annual gross monetary income from pensions in NIS for men who are either the respondent or spouse in the household	323	97,218	(102,324.0)
Owner	1 = own an apartment; 0 = otherwise	850	73%	(44.3%)
Books	1 = At least one book at the home library; 0 = no books at the home library	850	98%	(14.0%)
Car	1 = own a car; 0 = otherwise	850	65%	(47.7%)
Age	Age in years	850	75	(4.5)
Academic	1 = Formal academic education with BA, MA or Ph.D. diploma; 0 = otherwise	850	34%	(47.4%)
HHSIZE	Number of persons in household	850	2	(1.1)
Single	1 = Single; 0 = otherwise	850	2%	(13.6%)
Married	1 = Married; 0 = otherwise	850	75%	(43.3%)
Divorced	1 = Divorced; 0 = otherwise	850	8%	(27.7%)
Widow	1 = Widow; 0 = otherwise	850	15%	(35.4%)
Immigrant	1 = Immigrant; 0 = otherwise	850	0.696	(46.0%)
IMM_EUROPE_AMERICA	1 = Immigrant from European or American countries; 0 = otherwise	850	47%	(49.9%)
IMM_ASIA_AFRICA	1 = Immigrant from Asian or African countries; 0 = otherwise	850	23%	(41.8%)
IMM_EUROPE_AMERICA_PER	1 = Immigrant from European or American countries only for the group of immigrants; 0 = otherwise	592	68%	(46.9%)
OVERALL_HEALTH	1 = Self-reporting of good overall health conditions; 0 = otherwise	850	55%	(49.8%)

*Note*. The sample includes panel of 921 (850) females × years (males × years) belonging to 507 (466) households, where the age of female (male) members were restricted to be above 67 years, participating in the 2015-2016 longitudinal survey carried out by the Israeli Central Bureau of Statistics. The lower age bound is based on the retirement age from the workforce, which is 67 years for males. NIS is the local Israeli currency, where 1 NIS roughly equals $0.25. Standard deviations are given in parentheses.

**Table 9:** Pearson correlation matrix for females.

	BMI30	INCPENS	Books	Academic
BMI30	1.0000			
	[921]			
INCPENS	−0.1355^*∗∗∗*^	1.0000		
	(<0.0001)			
	[921]	[921]		
Books	−0.0158	0.0571	1.0000	
	(0.6318)	(0.1172)		
	[921]	[921]	[921]	
Academic	−0.0818^*∗∗*^	0.1750^*∗∗∗*^	0.0829^*∗∗*^	1.0000
	(0.0130)	(<0.0001)	(0.0119)	
	[921]	[921]	[921]	[921]

**Table 10:** Pearson correlation matrix for males.

	BMI30	INCPENS	Books	Academic
BMI30	1.0000			
	[850]			
INCPENS	0.0196	1.0000		
	(0.5680)			
	[850]	[850]		
Books	−0.0478	0.0339	1.0000	
	(0.1635)	(0.3239)		
	[850]	[850]	[850]	
Academic	−0.1294^*∗∗∗*^	0.1336^*∗∗∗*^	0.0674^*∗∗*^	1.0000
	(0.0002)	(0.0001)	(0.0496)	
	[850]	[850]	[850]	[850]

*Note*. *p* values are given in brackets. Number of observations is given in square brackets. ^*∗∗*^ *p* < 0.05 and ^*∗∗∗*^ *p* < 0.01 for difference from zero correlation.

**Table 11:** Random-Effect Regression 2015-2016: stratification by gender.

Variables	(1)	(2)	(3)	(4)
	Full	Stepwise	Full	Stepwise
	BMI30	BMI30	BMI30	BMI30
Constant	0.2245	0.2528^*∗∗∗*^	0.3330	0.2715^*∗∗∗*^
	(0.4940)	(<0.0001)	(0.3526)	(<0.0001)
INCPENS ÷ (10^4^)	−0.0041^*∗∗∗*^	−0.0052^*∗∗∗*^	0.0015	—
	(0.0021)	(0.0001)	(0.4225)	—
Owner	0.0031	—	−0.0342	—
	(0.9375)	—	(0.3460)	—
Books	0.0806	—	0.0013	—
	(0.2047)	—	(0.9875)	—
Car	−0.0460	—	0.0610^*∗*^	0.0641^*∗∗*^
	(0.1774)	—	(0.0897)	(0.0469)
Age	−0.0014	—	0.0003	—
	(0.7353)	—	(0.9494)	—
Academic	−0.0623^*∗*^	—	−0.1014^*∗∗*^	−0.0993^*∗∗*^
	(0.0966)	—	(0.0118)	(0.0106)
HHSIZE	0.0239	—	0.0059	—
	(0.1549)	—	(0.7551)	—
Married	0.0356	—	−0.0442	—
	(0.4045)	—	(0.7685)	—
Divorced	0.0925^*∗∗*^	—	−0.1623	—
	(0.0319)	—	(0.3060)	—
Widow	0.1128^*∗∗∗*^	0.0658^*∗∗*^	−0.1004	—
	(0.0048)	(0.0419)	(0.5222)	—
IMM_EUROPE_AMERICA	0.0162	—	−0.0767^*∗*^	−0.0899^*∗∗*^
	(0.7047)	—	(0.0821)	(0.0398)
IMM_ASIA_AFRICA	−0.0544	—	−0.1163^*∗∗*^	−0.1199^*∗∗*^
	(0.2377)	—	(0.0245)	(0.0200)
OVERALL_HEALTH	−0.0655^*∗∗*^	−0.0747^*∗∗∗*^	−0.0345	—
	(0.0190)	(0.0036)	(0.2439)	—
Gender	Females	Females	Males	Males
Age	Above 67	Above 67	Above 67	Above 67
Observations	921	921	850	850
R-squared	0.0569	0.0370	0.0560	0.0370
Households	507	507	466	466
Wald chi^2^: all coef. equals zero	47.25^*∗∗∗*^	28.59^*∗∗∗*^	25.09^*∗∗∗*^	19.67^*∗∗∗*^
Individual effect F-Statistics	6.79^*∗∗∗*^	6.98^*∗∗∗*^	4.58^*∗∗∗*^	4.69^*∗∗∗*^
Variance inflating factor (VIF)	2.34	1.07	2.78	1.25

*Note*. The table reports the estimation results of equation (3) for the group of retired women and men estimated via the random effect regression. The results of individual-effect F-statistics justify this procedure, which includes the individual effect dummies. The dependent variable is BMI30, a dummy variable, which equals one for Type I obesity (BMI ≥ 30) and zero otherwise. The stepwise procedure gradually omits variables with coefficients for whom *p* ≥ 0.05. Variance inflation factor (VIF) is a measure for collinearity among independent variables. VIF < 10 implies low collinearity. Robust *p* values are given in parentheses. ^*∗*^ *p* < 0.1. ^*∗∗*^ *p* < 0.05. ^*∗∗∗*^ *p* < 0.01.

**Table 12:** Random-Effect Regression 2015-2016: stratification by Overweight (BMI ≥ 25), Type I (BMI ≥ 30), and Type II (BMI ≥ 35) obesity among females above 67 years.

Variables	(1)	(2)	(3)	(4)	(5)	(6)
	Full	Stepwise	Full	Stepwise	Full	Stepwise
	BMI25	BMI25	BMI30	BMI30	BMI35	BMI35
Constant	1.0562^*∗∗∗*^	0.7178^*∗∗∗*^	0.2245	0.2528^*∗∗∗*^	0.0900	−0.0373
	(0.0036)	(<0.0001)	(0.4940)	(<0.0001)	(0.6601)	(0.3270)
INCPENS ÷ (10^4^)	−0.0084^*∗∗∗*^	−0.0095^*∗∗∗*^	−0.0041^*∗∗∗*^	−0.0052^*∗∗∗*^	−0.0015	—
	(0.0056)	(0.0014)	(0.0021)	(0.0001)	(0.1699)	—
Owner	−0.0538	—	0.0031	—	0.0414^*∗∗*^	0.0437^*∗∗∗*^
	(0.2784)	—	(0.9375)	—	(0.0169)	(0.0089)
Books	−0.0471	—	0.0806	—	−0.0287	—
	(0.5753)	—	(0.2047)	—	(0.3613)	—
Car	−0.0436	—	−0.0460	—	−0.0240	—
	(0.3183)	—	(0.1774)	—	(0.2806)	—
Age	−0.0033	—	−0.0014	—	−0.0014	—
	(0.4729)	—	(0.7353)	—	(0.5709)	—
Academic	−0.0204	—	−0.0623^*∗*^	—	0.0038	—
	(0.6753)	—	(0.0966)	—	(0.8731)	—
HHSIZE	0.0231^*∗*^	—	0.0239	—	0.0357^*∗∗*^	0.0353^*∗∗*^
	(0.0840)	—	(0.1549)	—	(0.0211)	(0.0201)
Married	−0.0388	—	0.0356	—	0.0433	−
	(0.7169)	—	(0.4045)	—	(0.3494)	−
Divorced	−0.1029	—	0.0925^*∗∗*^	—	0.1244^*∗*^	0.0983^*∗∗*^
	(0.4013)	—	(0.0319)	—	(0.0572)	(0.0340)
Widow	−0.0277	—	0.1128^*∗∗∗*^	0.0658^*∗∗*^	0.0906^*∗∗*^	0.0531^*∗∗*^
	(0.7978)	—	(0.0048)	(0.0419)	(0.0430)	(0.0377)
IMM_EUROPE_AMERICA	0.0406	—	0.0162	—	−0.0184	—
	(0.4103)	—	(0.7047)	—	(0.5098)	—
IMM_ASIA_AFRICA	−0.0468	—	−0.0544	—	−0.0379	—
	(0.4070)	—	(0.2377)	—	(0.1886)	—
OVERALL_HEALTH	−0.0535^*∗*^	−0.0632^*∗∗*^	−0.0655^*∗∗*^	−0.0747^*∗∗∗*^	−0.0245^*∗*^	−0.0282^*∗∗*^
	(0.0792)	(0.0275)	(0.0190)	(0.0036)	(0.0721)	(0.0288)
Gender	Female	Female	Females	Females	Female	Female
Age	Above 67	Above 67	Above 67	Above 67	Above 67	Above 67
Observations	921	921	921	921	921	921
R-squared	0.0485	0.0385	0.0569	0.0370	0.0853	0.0761
Households	507	507	507	507	507	507
Wald chi^2^: all coef. equals zero	27.53^*∗∗∗*^	17.73^*∗∗∗*^	47.25^*∗∗∗*^	28.59^*∗∗∗*^	25.54^*∗∗∗*^	19.41^*∗∗∗*^
Individual effect F-Statistics	5.54^*∗∗∗*^	5.55^*∗∗∗*^	6.79^*∗∗∗*^	6.98^*∗∗∗*^	6.79^*∗∗∗*^	6.51^*∗∗∗*^
Variance inflation factor (VIF)	2.34	1.06	2.34	1.07	2.34	1.10

*Note*. The table reports the estimation results of equation (3) for the group of retired women estimated via the random effect regression. The results of individual-effect F-statistics justify this procedure, which includes the individual effect dummies of households. The dependent variables are BMI25, BMI30, BMI35, and dummy variables, which equal one for Overweight (BMI ≥ 25), Type I (BMI ≥ 30), and Type II Obesity (BMI ≥ 35) and zero otherwise. The stepwise procedure gradually omits variables with coefficients for whom *p* < 0.05. Variance inflation factor (VIF) is a measure for collinearity among independent variables. VIF < 10 implies low collinearity. Robust *p* values are given in parentheses. ^*∗*^ *p* < 0.1. ^*∗∗*^ *p* < 0.05. ^*∗∗∗*^ *p* < 0.01.

**Table 13:** Random-Effect Regression 2015-2016: stratification by Overweight (BMI ≥ 25), Type I (BMI ≥ 30), and Type II (BMI ≥ 35) obesity among males above 67 years.

Variables	(1)	(2)	(3)	(4)	(5)	(6)
	Full	Stepwise	Full	Stepwise	Full	Stepwise
	BMI25	BMI25	BMI30	BMI30	BMI35	BMI35
Constant	1.4435^*∗∗∗*^	1.3911^*∗∗∗*^	0.3330	0.2715^*∗∗∗*^	0.0424	0.0256^*∗∗∗*^
	(0.0002)	(0.0001)	(0.3526)	(<0.0001)	(0.8479)	(0.0088)
INCPENS ÷ (10^5^)	0.0018	—	0.0015	—	0.0005	−
	(0.4404)	—	(0.4225)	—	(0.5758)	−
Owner	0.0025	—	−0.0342	—	0.0274^*∗∗*^	0.0255^*∗∗*^
	(0.9489)	—	(0.3460)	—	(0.0400)	(0.0431)
Books	−0.0535	—	0.0013	—	0.0467	—
	(0.1509)	—	(0.9875)	—	(0.5461)	—
Car	0.0050	—	0.0610^*∗*^	0.0641^*∗∗*^	0.0074	—
	(0.8735)	—	(0.0897)	(0.0469)	(0.4648)	—
Age	−0.0103^*∗∗*^	−0.0099^*∗∗*^	0.0003	—	−0.0004	—
	(0.0328)	(0.0450)	(0.9494)	—	(0.8297)	—
Academic	−0.0579	—	−0.1014^*∗∗*^	−0.0993^*∗∗*^	0.0225	—
	(0.2690)	—	(0.0118)	(0.0106)	(0.6095)	—
HHSIZE	0.0051	—	0.0059	—	0.0101	—
	(0.7859)	—	(0.7551)	—	(0.4697)	—
Married	0.0126	—	−0.0442	—	−0.0489	—
	(0.9057)	—	(0.7685)	—	(0.3894)	—
Divorced	−0.0515	—	−0.1623	—	−0.0538	—
	(0.6554)	—	(0.3060)	—	(0.3428)	—
Widow	−0.0101	—	−0.1004	—	−0.0504	—
	(0.9233)	—	(0.5222)	—	(0.3341)	—
IMM_EUROPE_AMERICA	0.0718	—	−0.0767^*∗*^	−0.0899^*∗∗*^	−0.0197	—
	(0.1637)	—	(0.0821)	(0.0398)	(0.4167)	—
IMM_ASIA_AFRICA	−0.0450	—	−0.1163^*∗∗*^	−0.1199^*∗∗*^	−0.0036	—
	(0.4567)	—	(0.0245)	(0.0200)	(0.8975)	—
OVERALL_HEALTH	0.0027	—	−0.0345	—	−0.0164	—
	(0.9232)	—	(0.2439)	—	(0.3300)	—
Gender	Males	Males	Males	Males	Males	Males
Age	Above 67	Above 67	Above 67	Above 67	Above 67	Above 67
Observations	850	850	850	850	850	850
R-squared	0.0255	0.00900	0.0560	0.0370	0.0157	0.00573
Households	466	466	466	466	466	466
Wald chi^2^: all coef. equals zero	13.48	4.020^*∗∗*^	25.09^*∗∗∗*^	19.67^*∗∗∗*^	8.487	4.091^*∗∗*^
Individual effect F-Statistics	7.13^*∗∗∗*^	7.07^*∗∗∗*^	4.58^*∗∗∗*^	4.69^*∗∗∗*^	13.38^*∗∗∗*^	11.06^*∗∗∗*^
Variance inflation factor (VIF)	2.78	1.00	2.78	1.25	2.78	1.00

*Note*. The table reports the estimation results of equation (3) for the group of retired men estimated via the random effect regression. The results of individual-effect F-statistics justify this procedure, which includes the individual effect dummies of households. The dependent variables are BMI25, BMI30, BMI35, and dummy variables, which equal one for Overweight (BMI ≥ 25), Type I (BMI ≥ 30), and Type II Obesity (BMI ≥ 35) and zero otherwise. The stepwise procedure gradually omits variables with coefficients for whom *p* < 0.05. Variance inflation factor (VIF) is a measure for collinearity among independent variables. VIF < 10 implies low collinearity. Robust *p* values are given in parentheses. ^*∗*^ *p* < 0.1. ^*∗∗*^ *p* < 0.05. ^*∗∗∗*^ *p* < 0.01.

## Data Availability

The data used to support the findings of this study are available from the corresponding author upon request.
